# Genetic Structure and Phylogeographic Divergence of *Thymallus brevicephalus* in the Ob‐Irtysh River Headwaters

**DOI:** 10.1002/ece3.70422

**Published:** 2024-10-11

**Authors:** Wenjie Peng, Haoxiang Han, Bo Ma

**Affiliations:** ^1^ Heilongjiang River Fishery Research Institute of Chinese Academy of Fishery Sciences Harbin China; ^2^ Dalian Ocean University Dalian China; ^3^ Scientific Observing and Experimental Station of Fishery Resources and Environment in Heilongjiang River Basin Ministry of Agriculture and Rural Affairs Harbin China

**Keywords:** Altai Mountains, biogeography, conserve genet, Irtysh River, *Thymallus*

## Abstract

Clarifying the genetic structure and population history of a species can reveal the impacts of historical climate and geological changes, providing critical insights for developing effective conservation strategies for ecologically significant fish. The Markakol grayling (*Thymallus brevicephalus*), an endangered species found in the Altai‐Sayan Mountain region of Central Asia, serves as an ideal model for studying these factors. In this study, populations of a grayling (*Thymallus*) species discovered in the upper Irtysh River headwaters in Xinjiang, China, were analyzed to assess genetic diversity and population structure. Mitochondrial DNA sequences (cytochrome b and control region), along with 10 microsatellite markers, were used to examine genetic variation. Phylogenetic and genetic distance analyses confirmed the species, long misidentified as Arctic grayling (*T. arcticus*), as *T. brevicephalus*. This species can be divided into two distinct geographic groups: eastern and western, with the Crane River acting as the boundary. The divergence between these groups likely corresponds to refugia formed during the Pleistocene glaciation of the Altai Mountains, approximately 0.48 MA (million years ago) (range: 0.30 to 0.71 Ma). High haplotype diversity (Hd > 0.5) and low nucleotide diversity (π < 0.005) suggest that, despite the species' genetic richness, *T. brevicephalus* remains vulnerable to genetic drift, which could threaten its long‐term survival. This vulnerability may stem from inbreeding within small refugial populations during the glacial period, followed by gradual population expansion. Our study offers novel insights into grayling populations, with results that have direct implications for management by serving as a tool for the identification of conservation units.

## Abstract 2

This study reveals the impact of past climate events on a fish species (*Thymallus brevicephalus*) in China. Genetic analysis suggests the species split into eastern and western populations during a past glaciation event. While the fish show genetic diversity, specific management plans are needed to conserve both eastern and western populations due to their unique evolutionary histories.

## 1 Introduction

Environmental changes, including geological and climate shifts, can drastically alter the habitats and distribution patterns of species, impacting their survival and reproduction (Weigelt et al. 2016; Liu et al. 2011; Wanghe et al. 2017). Because the distributions of freshwater fish are affected by watercourse constraints, geological and climatic changes to these waterways can lead to species differentiation through isolation, or range expansion through increased connectivity of drainage systems. To maintain species' genetic integrity, understanding the interactions among geological and climatic changes, as well as drainage systems, and their relationships with freshwater fish species, is crucial. This understanding helps elucidate the mechanisms affecting their diversity and is essential for devising appropriate management and conservation strategies.

The Altai Mountains, located at the convergence of China, Kazakhstan, Mongolia, and Russia, have undergone several phases of mountain‐building and uplift, particularly during the Pleistocene to Holocene epochs. These geological changes, coupled with climatic fluctuations, have reshaped the region's landscape and directly influenced the biodiversity within the mountain range (Huangfu et al. 2023; Zang et al. 2015; Zhao et al. 2013). The Irtysh River, originating on the southern slopes of the Altai, features a unique comb‐shaped drainage system, where tributaries from the north and south merge to form the main channel. After passing through Lake Zaysan, the river flows northward, joining the Ob River to form the Ob‐Irtysh system, which ultimately drains into the Arctic Ocean. The diverse aquatic ecosystems along this river system have faced increasing pressures from anthropogenic activities over the past 50 years, including overfishing and the construction of downstream hydroelectric facilities. These activities have already led to the regional extinction of *Stenodus leucichthys* in China, a migratory salmonid species once abundant in the upper Irtysh River during the 1960s (Freyhof and Emma 2011; Poursaeid and Falahatkar 2012).

Given this history of extinctions and habitat degradation, other migratory species, such as *Thymallus* spp., are at risk of facing similar threats if effective conservation measures are not implemented. Protecting these species is critical for preserving both regional genetic diversity and the ecological balance of the Irtysh River system. *Thymallus*, widely distributed across Eurasia and North America, is known for its conservative reproductive behavior, with individuals repeatedly spawning in the same locations and exhibiting limited migratory capabilities (Gönczi 1989; Northcote 1995; Nykänen, Huusko, and Mäki‐Petäys 2001). Consequently, the distribution patterns of Grayling species are highly unique. Within a single river system, *Thymallus* populations often display high levels of genetic differentiation, as reported for the Amur river (Froufe et al. 2003; Ma et al. 2012; Weiss, Secci‐Petretto et al. 2020), Yenisei river (Andrushchenko et al. 2023; Knizhin, Bogdanov, and Vasil'eva 2006; Knizhin and Weiss 2009), and Lena river (Knizhin, Kirillov, and Weiss 2006; Weiss et al. 2006). This phenomenon can even extend into a single lake, such as, for example, the Hoton Nur Lake in western Mongolia (Slynko, Mendsaykhan, and Kas'anov 2010) and Lake Saimaa in eastern Finland (Koskinen, Piironen, and Primmer 2001; Koskinen, Knizhin et al. 2002). Significant morphological differences also exist among *Thymallus* in Lake Baikal (Knizhin et al. 2006; Knizhin, Weiss, and Sušnik 2006). *Thymallus* serves as a model organism for studying how geography and past environmental changes have shaped their genetic diversity. This knowledge not only informs research across various fields but also empowers us to develop more effective protection strategies for these ecologically important fish.

The incomplete understanding of the diversity, geographical distribution, and biology of Grayling species, particularly in the Altai Mountains, obscures the relationship between geological and climatic changes and this diversity, hindering effective conservation efforts. Northwest of this mountain range, the Upper Ob grayling (*T. nikolskyi*) inhabits the Ob River, which is hydrologically linked to the Irtysh River. To the south, the Markakol grayling (*T. brevicephalus*) inhabit Lake Markakol and its surrounding water systems, and it is considered a sister species to the Mongolian grayling (*T. brevirostris*) in the northern part of the Altai Mountains, despite significant morphological differences (Weiss, Grimm et al. 2020; Weiss et al. 2021; Knizhin et al. 2008). Additionally, grayling species occur in the upper Irtysh River headwaters in China, where they are typically referred to as Arctic grayling (*T. arcticus*) (Guo, Zhang, and Cai 2012; Liu et al. 2016; Ren, Guo, and Zhang 2002).

To comprehend the genetic structure and population history of the grayling species in upper Irtysh River in China and to provide data supporting grayling conservation efforts in the region, mitochondrial data were collected from grayling species inhabiting the upper Irtysh River in China. The original data were combined with mitochondrial data obtained from the literature for molecular phylogenetic analysis. To better understand the distribution patterns and population genetic structure of these fish in these mountains, 10 polymorphic microsatellite loci were used to investigate genetic diversity and population structure. In addition, we considered geological and climatic events to further explore the evolutionary history of this taxon in the region.

## 2 Materials and Methods

**FIGURE 1:**
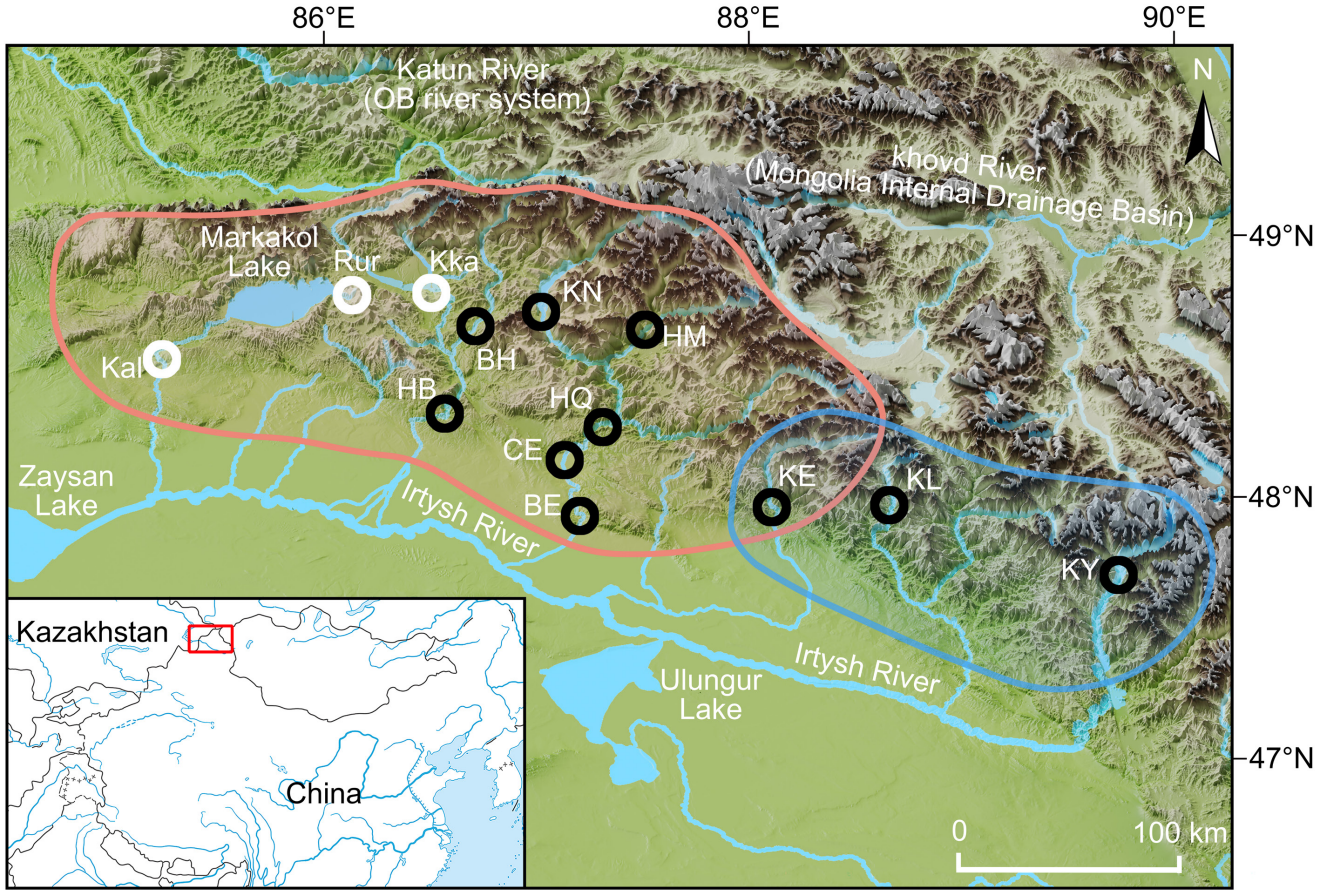
Study area; lower left, the East Asia, indicating study area location. Sampling points are differentiated by color: Black, samples from the upper Irtysh River within China; white, samples from Kazakhstan territory along the upper Irtysh River (from the literature). The figure also includes the approximate distribution ranges of Grayling species: Red represents *T. brevicephalus* group A (western); blue represents *T. brevicepahlus* group B (eastern).

Fishes (*n* = 161) were captured in October 2019 from 10 locations in the Irtysh River basin, Xinjiang, China (Figure 1, Table 1). All captured fish were briefly anesthetized with an MS‐222 bath at a concentration of 60.0 mg/L, and fin clipping was performed as quickly as possible. The fish were immediately released after sampling, followed by subsequent monitoring to ensure their full recovery. Genomic DNA was extracted using an Ezup Column Animal Genomic DNA Extraction Kit from Sangon Biotech (Shanghai) Co., Ltd.

**TABLE 1:** Sample locations including sample location, sample population code, drainage basin, number of individuals screened for both mtDNA and microsatellite loci, geographical coordinates s, and referenced GenBank accession numbers for *Thymallus* samples in this study.

Sample location	Sample code	Number of individuals		Lat. (N)	Long. (E)	GenBank numbers
		mtDNA	Microsatellite			
Kayiertesi River	KY	25	12	47°38′31″	89°44′55″	
Karaertis River	KL	25	45	47°58′53″	88°40′10″	
Crane River	KE	16	14	47°54′38″	88°7′25″	
Hongqi Reservoir	HQ	15	—	48°4′33″	87°7′38″	
Burqin River	BE	15	—	47°47′26″	87°5′49″	
Chonghuer Reservoir	CE	15	—	48°4′33″	87°7′38″	
Hemu River	HM	13	—	48°40′14″	87°32′57″	
Kanas River	KN	15	27	48°47′8″	87°1′56″	
Akkaba River	BH	7	—	48°30′11″	86°36′9″	
Kara‐Kaba River	HB	15	13	48°43′17″	86°46′23″	
Kara‐Kaba River	Kka	14 (CR only)	—	48°48′	48°48′	MN030584–MN030597
Urunkhaika River	Rur	12 (CR only)	—	48°46′07	48°46′07	MN030598–MN030609
Kaldzhir River	Kal	6 (CR only)	—	48°25′	48°25′	MN030578–MN030583

To amplify cytochrome b (Cyt b) gene sequences, we used L14724 (5'‐GACTTGAAAAACCACCGTTG‐3′) and H15915 (5'‐CTCCGATCTCCGGATTACAAGAC‐3′) primers. For the control region (CR) gene sequence, we used D‐loop‐F (5'‐ACCCCTGGCTCCCAAAGC‐3′) and D‐loop‐R (5'‐ATCTTAGCATCTTCAGTG‐3′) primers. After PCR amplification, the products were purified and sequenced by Sangon Biotech (Shanghai) Co., Ltd.

We used published primers originally developed for grayling (Table S1) (Weiss, Grimm et al. 2020) and performed PCR reactions. Fragment sizes were determined using the 3730xl (ABI) and analyzed with GENEMAPPER software v5.0 (Applied Biosystems).

### 2.1 mtDNA Data Analysis

The cytochrome b (Cyt b) combined with control region (CR) yielded a total of 161 sequences. Thirty‐two sequences of *T. brevicephalus* were included in all CR sequences analyses conducted in this study (GenBank accession no. MN030578–MN030609). Multiple sequence alignment was performed using the ClustalW method and inter‐population genetic distances based on the uncorrected p‐distances were estimated in MEGA 11.0.13. (Larkin et al. 2007; Tamura, Stecher, and Kumar 2021), and haplotype analysis and nucleotide diversity was computed using DnaSP v6.12.03 (Rozas et al. 2017). The analysis of molecular variance (AMOVA) computations was performed using Arlequin 3.5 to detect genetic variation among geographic populations or groups and to calculate genetic variation indices (Excoffier and Lischer 2010). The significance of covariance at different levels of genetic structure was tested through 1000 resampling iterations.

Phylogenetic analysis was conducted using CR and the concatenated dataset (5'‐Cyt b + CR‐3′) with Maximum Likelihood (ML) estimation in raxmlGUI 2.0 (Edler et al. 2021) and Bayesian Inference (BI) in MrBayes 3.2.7 (Ronquist et al. 2012). The best nucleotide substitution models for Bayesian Inference (BI) and Maximum Likelihood (ML) analyses in CR and Cyt b + CR phylogenetic analysis were identified as HKY + G using MrModeltest 2 and MEGA 11.0.13 (Nylander et al. 2004; Tamura, Stecher, and Kumar 2021). Mongolian grayling (*T. brevirostris*) (GenBank accession no. MH027384, IMT063033, NC027412, KJ866486), Upper Ob grayling (*T. nikolskyi*) (MT063030, MT063029, NCO56308), Baikal black grayling (*T. baicalensis*) (MT063022, MT063023), Chovsgul grayling (*T. nigrescens*) (NC056307, MTO63028), Lower Amur grayling (*T. tugarinae*) (KY078217, KY078218), *Coregonus muksun* (NCO28593) and *Hucho taimen* (MK258080) were used as outgroups.

Haplotype networks inference methods used TCS for the CR and Cyt b + CR were constructed using PopArt1.7 (Leigh and Bryant 2015). In the CR haplotype networks, four sequences of Mongolian grayling (*T. brevirostris*) from northeastern Altai Mountains in Mongolia (EU676266, EU676267, EU676274, EU676281) were included to assess the phylogenetic relationships between the study area and adjacent grayling species.

To estimate divergence times between species, a time‐calibrated Cyt b + CR phylogeny was constructed using BEAUti in BEAST 1.10.4. This BI phylogenetic analysis used the HKY + G substitution model. A molecular clock was modeled using uncorrelated relaxed molecular clock priors, specifically lognormal distributions (Suchard and Rambaut 2009). The Birth–Death speciation model was selected as the tree prior, due to the dataset encompassing both intra‐ and inter‐specific relationships. The mtDNA molecular clock calibration for Salmoniformes species was set at 1% per Ma (million years ago) as an overall mutational rate applied directly in BEAUti, with a relative death rate following a normal distribution (mean 0.01, SD 0.002). Two divergence times were specified: the most recent common ancestor of Salmoniformes around 50 Ma (lognormal distribution, offset 50, mean 10, SD 1) and the period around 0.13 Ma when *Thymallus baicalensis* entered Lake Baikal (normal distribution, mean 0.12, SD 0.1) (Koskinen, Nilsson et al. 2002; Weiss et al. 2021). Iterations were run 30 × 10^6^ times, with sampling conducted every 3000 iterations. Generated data were assessed for convergence and effective sample sizes (> 200) using Tracer v1.7 (Rambaut et al. 2018). The final tree was constructed using TreeAnnotator v1.10.4 (Dellicour et al. 2021), and optimized on the iTOL (Letunic and Bork 2021).

Tajima's D test and Fu's Fs test in Arlequin 3.5 software (Excoffier and Lischer 2010), as well as the sum of squared deviation (SSD) and Harpending's raggedness index (*r*), were used to infer population historical demography. Mismatch distributions were analyzed using DnaSP v6.12.03 (Rozas et al. 2017). BEAST (Drummond et al. 2012) using Bayesian Skyline Plot (BSPs) was used to infer historical demography, using parameter settings consistent with those used in divergence time analysis and under the same clock mutation rate. Finally, Tracer 1.7 software was used to visualize and edit the historical demography of effective population size (Rambaut et al. 2018).

### 2.2 Nuclear Microsatellite Genotyping and Data Analysis

Preliminary analysis indicates that Grayling populations in the upper Irtysh River are conspecific. We used 10 microsatellite loci markers for supplementary phylogenetic reconstruction to further understand genetic relationships between populations. Using FSTAT v2.9.3.2 (Goudet 2001), we calculated the number of alleles per locus, deviations based on the FIS values for Hardy–Weinberg equilibrium, and deviations from Linkage Equilibrium. ARLEQUIN v3.5.2.2 was used to calculate observed and expected heterozygosity, as well as to perform analyses based on the infinite allele model (FST) (Excoffier and Lischer 2010). GenAlEx was used to calculate Nei's genetic distance among populations. Principal coordinates analysis (PCoA) was performed based on the genetic distances between individuals (Peakall and Smouse 2012). A clustering tree was constructed based on Nei's genetic distance using MEGA (Tamura, Stecher, and Kumar 2021).

Overall genetic structure was assessed using the Bayesian clustering method in STRUCTURE v2.3 (Porras‐Hurtado et al. 2013). Prior values of *K* (number of populations) were assumed between 1 and 5. STRUCTURE was run for 100,000 iterations, with the first 50,000 iterations discarded as burn‐in, and five independent MCMC replicates were performed for each *K* value. The Delta *K* statistic method (Earl and vonHoldt 2012) were used to determine the most suitable *K* value. After obtaining output results, K matrices from multiple runs were merged using CLUMPP (Jakobsson and Rosenberg 2007); the visualization was optimized using Distruct (Rosenberg 2004).

To further decipher the potential genetic structure of *Thymallus* in the upper Irtysh River, a cluster analysis was conducted using the stochastic optimization method in BAPS 6.0 (Corander et al. 2008). BAPS analysis parameters were initially set to *K* = 1 to 5, with each *K* value repeated 10 times; the maximum Log marginal likelihood [Log(ML)] value determines the best *K*. For population admixture analysis, the number of iterations was set to 1000; other settings remained at default values.

## 3 Results

### 3.1 Phylogenetic Analysis

The alignment of the CR region was 1072 bp, with 14 variable sites and 13 parsimony sites. Upon combining our sequences with CR sequences from *T. brevicephalus*, a total of 193 sequences were generated, with 17 variable sites, 14 of which were parsimony sites, resulting in 19 CR haplotypes. The Cyt b alignment was 1105 bp, with 4 variable sites, all being parsimony sites, resulting in 4 Cyt b haplotypes.

Concatenating each sample into a 2177 bp sequence in the format of 5'‐Cyt b + CR‐3′ for phylogenetic analysis yielded 17 variable sites, all of which were parsimony informative. The concatenated dataset resulted in a total of 26 mtDNA haplotypes, Except for the KL population, all groups showed results for CR and Cyt b + CR indicating high haplotype diversity (Hd > 0.5) and low nucleotide diversity (π < 0.005) (Table 2).

**TABLE 2:** Genetic diversity indices for 10 populations of *Thymallus brevicephalus* based on CR, and concatenated Cyt b + CR sequence data sets.

Sequence	Population	*n*	*S*	*h*	Hd	*k*	π
CR	KY	25	4	5	0.607	1.02	0.00095
	KL	25	2	3	0.477	0.5	0.00047
	KE	16	9	7	0.825	3.09167	0.00288
	HQ	15	6	5	0.819	2.41905	0.00226
	BE	15	6	5	0.79	2.4381	0.00227
	CE	15	6	5	0.81	2.68571	0.00251
	HM	13	5	4	0.744	1.97436	0.00184
	KN	15	7	8	0.848	2.34286	0.00219
	BH	7	2	3	0.762	0.95238	0.00089
	HB	15	4	4	0.686	1.25714	0.00117
	Total	161	14	18	0.884	3.60621	0.00336
Cyt b + CR	KY	25	4	5	0.607	1.02	0.00095
	KL	25	2	3	0.477	0.5	0.00047
	KE	16	9	7	0.825	3.09167	0.00288
	HQ	15	6	5	0.819	2.41905	0.00226
	BE	15	6	5	0.79	2.4381	0.00227
	CE	15	6	5	0.81	0.68571	0.00251
	HM	13	5	4	0.744	1.97436	0.00184
	KN	15	7	8	0.848	2.34286	0.00219
	BH	7	2	3	0.762	0.95238	0.00089
	HB	15	5	4	0.686	1.50476	0.00069
	Total	161	17	26	0.907	4.40357	0.00202

Abbreviations: *h*, number of haplotypes; Hd, haplotype diversity; *K*, number of nucleotide differences; *n*, number of samples; *S*, number of segregating sites; π, nucleotide diversity.

Based on the analysis of genetic differences (uncorrected p‐distances) shown in Figure S1, the grayling from the upper Irtysh River in China exhibits a very small genetic distance (CR minimum distance of 0.0008) compared to *T. brevicephalus* from Kazakhstan. This suggests that the grayling in the upper Irtysh River are likely the same species (synonymous) as *T. brevicephalus*. In contrast, the mean divergence distances between *T. brevicephalus* and *T. brevirostris* show a greater distance (CR: 0.0053, Cyt b + CR: 0.0063). Within *T. brevicephalus*, the CR distance between eastern populations (KL and KY) is 0.0009, and the Cyt b + CR distance is 0.0006. For the populations west of KE (KE, HB, BH, KN, CE, BE, HM, HQ), the average CR distance is 0.0023, and the Cyt b + CR distance is 0.0016. The average distance between these two geographic groups is 0.0051 for CR and 0.0029 for Cyt b + CR. The gradient from dark blue to light blue in the images reflects the clear structural differences between species and geographic groups. The AMOVA results (Table 3) for CR and Cyt b + CR indicated that the variation is almost equally divided between among populations and within populations. After dividing the data into two geographic groups, the variation between the two regions accounted for the largest proportion (CR: 63.82%, Cyt b + CR: 56.98%).

**TABLE 3:** Molecular variance (AMOVA) for *T. brevicephalus* population genetic variation based on Cyt b, CR and concatenated Cyt b + CR sequence data sets, and nuclear DNA microsatellite variation.

Source of variation	Variance component	Percentage of variation	Fixation index
**CR**			
Among populations	1.0214	53.49	—
Within populations	0.88828	46.51	FST = 0.535*
Between regions (A/B)	1.82666	63.82	—
Among populations within regions	0.14259	4.99	—
Within populations	0.88828	31.09	FST = 0.689*
**Cyt b + CR**			
Among populations	1.13727	49.01	—
Within populations	1.18318	50.99	FST = 0.490*
Between regions (A/B)	1.87709	56.98	—
Among populations within regions	0.2342	7.11	—
Within populations	1.18318	35.91	FST = 0.641*

* P < 0.001.

Two phylogenetic methods were used to construct CR and Cyt b + CR trees, both of which resulted in identical topological structures. Images illustrate node‐support values obtained from tree‐building methods. Following divergence of *T. tugarinae* in the easternmost Eurasian continent, two Grayling species within the Baikal Lake region split. The three grayling species from the Altai–Sayan Mountains form a branch. This also confirms that the grayling species in the upper Irtysh River in China are likely synonymous with *T. brevicephalus* (Figure S2), with the merged *T. brevicephalus* population showing a clear internal geographical structuring pattern. This species then clusters with Mongolian grayling (*T. brevirostris*) from the north of the Altai Mountains. These findings receive robust support from bootstrap values (Figures S2 and S3).

**FIGURE 2:**
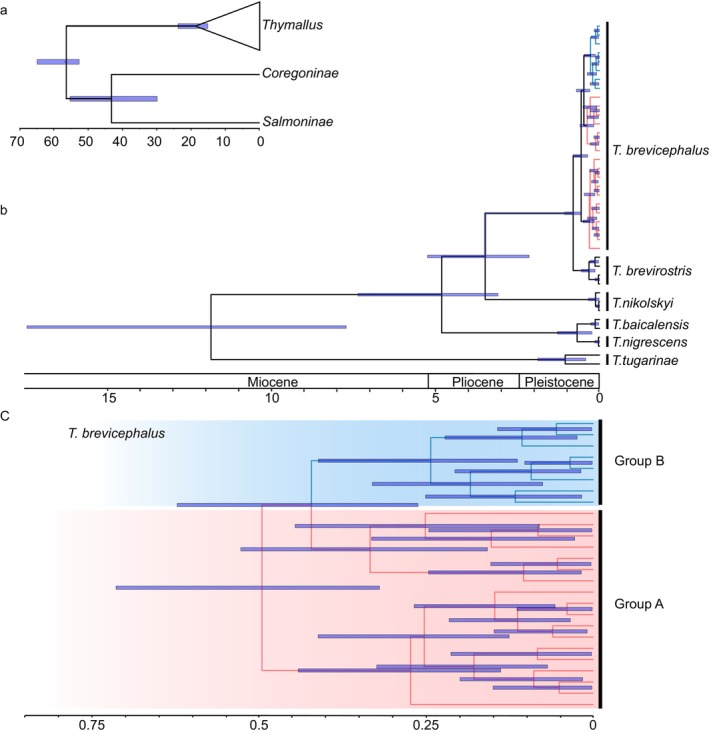
Time‐calibrated Bayesian phylogenetic tree based on mtDNA CR region sequences. Blue shaded bars indicate 95% credibility interval intervals for node ages; the scale represents millions of years from prese, (a) Within the Salmonidae family, (b) Among *Thymallus* species, (c) Within *T. brevicephalus*.

According to the time‐calibrated Cyt b + CR phylogenetic estimation, the divergence time between *T. brevirostris* and *T. brevicephalus* is estimated at 0.81 Ma (0.57–1.08) (Figure 2b). The time to the most recent common ancestor for the group A and B within *T. brevicephalus* is estimated to be 0.48 Ma (0.30–0.71), with a divergence time of 0.39 Ma (0.18–0.60) within group A and 0.28 Ma (0.13–0.47) within group B (Figure 2c).

**FIGURE 3:**
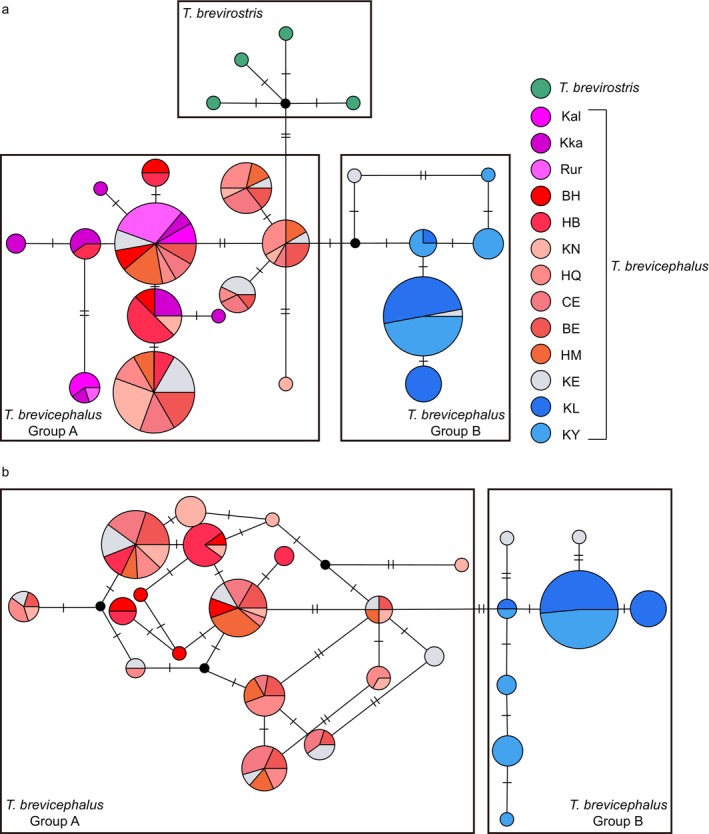
(a) Control Region (CR) haplotype network for *T. brevirostris* and *T. brevicephalus*; (b) Cyt b + CR haplotype network for Irtysh River grayling. Haplotype circle diameter represents the observed number of individuals. Colors correspond to different regions; black circles represent unobserved intermediate haplotypes. Small bars crossing lines indicate numbers of mutational steps between adjacent haplotypes.

The CR haplotype network exhibits clear geographical structuring (Figure 3a). Except for the KE population (gray), which shares haplotypes in both geographic groups A and B, the two geographic groups show distinct boundaries. Based on the geographical distribution of individuals, all individuals in geographic group A originate from west of KE, while all individuals in geographic group B originate from east of KE. Each side has a central haplotype that may represent the ancestral haplotype of their respective group. The CR haplotype network shows that at the species level, the closest haplotype distance between *T. brevirostris* and *T. brevicephalus* is only three mutational steps, indicating a close genetic relationship between the two species. To clarify the geographic structure of *T. brevicephalus*, we conducted a haplotype network analysis combining Cyt b and CR. The results showed a geographic structure similar to that of the CR results (Figure 3b), but with the KE population sharing haplotypes only with individuals from group A. The combined haplotype network provided a clearer distinction between the eastern and western branches.

**FIGURE 4:**
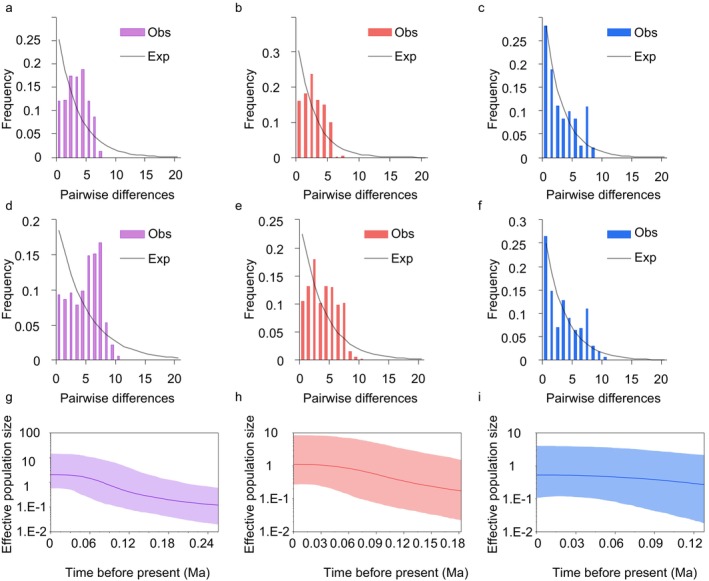
Mismatch distributions and Bayesian Skyline Plots (BSP) in *T. brevicephalus*. Mismatch distributions based on CR sequences (a–c): (a) total haplotypes, (b) western group, (c) eastern group; Mismatch distributions based on Cyt b + CR sequences (d–f): (d) total haplotypes, (e) group A, (f) group B; columnar lines represent observed distribution curves; solid lines represent expected distribution curves. Bayesian Skyline Plots (BSP) based on Cyt b + CR sequences (g–i): (g) total haplotypes, (h) group A, (i) group B; Axes: X, time in millions of years before present; Y (log scale), estimated effective population size (Ne) of females multiplied by generation time. The solid line indicates the median population size estimate, and the 95% credibility interval is depicted as a semi‐transparent region surrounding the outer boundary.

Mismatch distribution analysis based on CR and Cyt b + CR sequences indicate a unimodal distribution for *T. brevicephalus* as a whole (Figure 4a,d) and for group A (Figure 4b,e). BSP analysis (Figure 4g) reveals sustained expansion events for both the entire *T. brevicephalus* population and group A over an extended period (0.26–0.06 Ma). Negative Fu's Fs and Tajima's D values also corroborate these population expansion events (Table 4).

**TABLE 4:** Mismatch distribution and neutrality test for five populations of *T. brevicephalus* based on the CR and concatenated Cyt b + CR sequence data sets.

Sequence	Haplotype lineage	SSD	*r*	Tajima's D	Fu's *F*s
CR	Total	0.0045	0.0123	−1.011	−5.975
	Lineage A	0.0037	0.0214	−1.280	−2.848
	Lineage B	0.0001	0.0527	−0.141	−1.468
Cyt b + CR	Total	0.0093	0.0179	1.240	−5.066
	Lineage A	0.0068	0.0185	0.811	−4.319
	Lineage B	0.0091	0.0897	−0.829	−0.745

### 3.2 Genetic Diversity and Genetic Structure of Microsatellite Data

Microsatellite Pairwise FST values (Table 5) range from 0.021 to 0.084, with all results showing significant differences (*p* < 0.01) except for KN/KE (0.021). The genetic distances between KN and HB in the western region range from 0.021 to 0.062, while the distance between KL and KY in the eastern region is 0.041, all indicating moderate to low levels of differentiation. KE exhibits moderate differentiation from both the eastern and western geographic groups (0.056–0.075), while moderate levels of differentiation are observed between eastern and western populations (0.062–0.084). These results are consistent with those based on Nei's unbiased genetic distances.

**TABLE 5:** Matrix of pairwise FST values (below diagonal) and Nei's unbiased genetic distances (above diagonal) among five *T. brevicephalus* populations based on microsatellite data.

	HB	KN	KE	KL	KY
HB		0.388	0.550	0.692	0.752
KN	0.046*		0.073	0.457	0.575
KE	0.062*	0.021		0.554	0.691
KL	0.070*	0.044*	0.056*		0.244
KY	0.084*	0.062*	0.075*	0.041*	

* *p* < 0.01.

The average number of alleles per locus (Na) across the five populations is 9.56. The mean allelic richness per sample (AR) is 3.53. The average expected heterozygosity for all sampling locations is relatively high, ranging 0.748 (KN) to 0.860 (KY). Average observed heterozygosity for all sampling locations is also high, ranging 0.623 (KL) to 0.677 (KE). Mean expected heterozygosity values are higher than mean observed heterozygosity values, the FIS value ranging from 0.107 to 0.210 indicates a moderate level of inbreeding within the population. This suggests that random mating among individuals within the population is not entirely occurring, but there is a certain degree of mating among relatives (Table 6).

**TABLE 6:** Genetic statistical summaries of microsatellite analysis for five *T. brevicephalus* populations.

Sample code	NA	AR	HO	HE	FIS
HB	10	2.96	0.64615	0.82154	0.169
KN	12	4.77	0.66666	0.87592	0.210
KE	7.7	4.92	0.67727	0.84642	0.194
KL	11.6	3.00	0.62321	0.80107	0.168
KY	6.5	2	0.58333	0.7721	0.107

**FIGURE 5:**
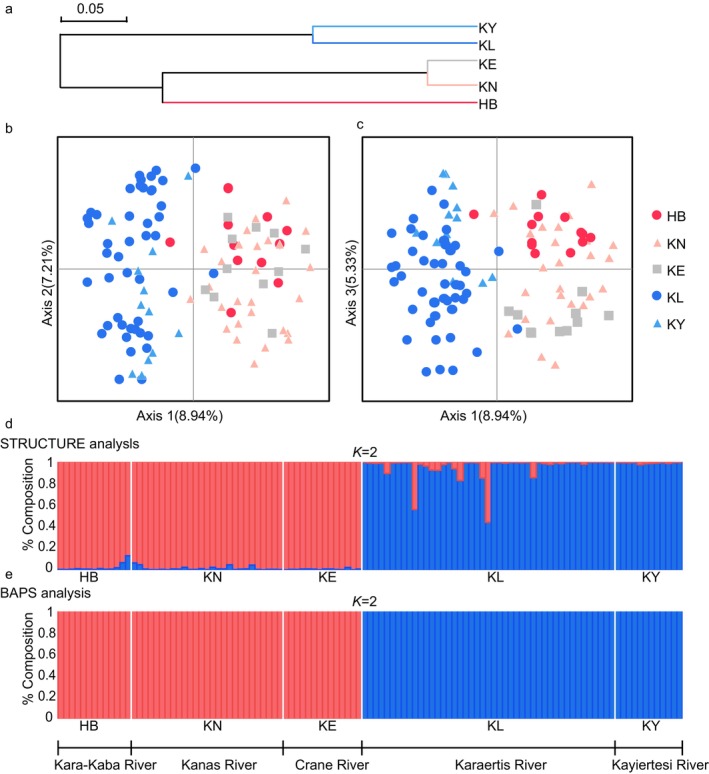
Genetic relationships among *T. brevicephalus* populations HB, KN, KE, and KL in the upper Irtysh River using 10 microsatellite loci. (a) NJ clustering tree based on Nei's unbiased genetic distances; (b) The first two axes of the Principal Coordinate Analysis (PCoA) based on pairwise genetic distances between individuals; (c) The first and third axes of the Principal Coordinate Analysis (PCoA) are based on pairwise genetic distances between individuals; (d) allocation test histogram using STRUCTURE (*K* = 2); (e) allocation test histogram using BAPS (*K* = 2), Each individual is represented by a vertical colored line, with each color corresponding to a genetic cluster.

The Neighbor‐Joining clustering tree based on Nei's unbiased genetic distances (Figure 5a) also reveals a clear geographic structure. The five populations form two branches, with KE, KN, and HB from the west clustering together, and the eastern populations, KL and KY, forming another distinct cluster. The PCoA diagrams (Figure 5b,c) illustrate the divergence between the East and West geographic groups along the first (8.94%) and second (7.21%) axes, as well as along the first (8.94%) and third (5.33%) axes. Individuals from HB, KN, and KE are primarily concentrated in quadrants 1 and 2, while KL and KY individuals are predominantly clustered in quadrants 3 and 4. These results show that KE groups together with the western populations, forming two distinct lineages and a clear geographic pattern.

STRUCTURE analysis indicates that the highest posterior probability was observed at ΔK = 2. Consequently, we present the clustering model (Figure 5d), revealing that individuals belonging to the western populations of *T. brevicephalus* (HB, KN, KE) form one cluster, while individuals from the eastern populations (KL, KY) constitute another, with membership coefficients > 96.4% for all individuals. BAPS analysis reveals the Log (ML) statistic to peak at *K* = 2. the overall pattern (Figure 5e) of differentiation is consistent with the STRUCTURE model. This pattern aligns with conclusions drawn from clustering tree and PCoA analyses, supporting the existence of two geographically structured groups among the five populations of *T. brevicephalus*: KL and KY as the eastern group, and KE, HB, and KN as the western group.

## 4 Discussion

### 4.1 Genetic Structure and Phylogeographic Patterns of *T. brevicephalus* in the Upper Irtysh River

This study suggests that the Arctic grayling (*T. arcticus*) in the upper Irtysh River in China should be classified as Markakol grayling (*T. brevicephalus*). Combining geographical distribution and divergence time, *T. brevicephalus* and Mongolian grayling (*T. brevirostris*) were geographically isolated on the northern and southern sides of the Altai Mountains at approximately 0.81 MY. This isolation is attributed to geological changes induced by the second uplift of the Altai Mountains since the Middle Pleistocene (~1.00 MY) (Bolikhovskaya and Shunkov 2014; Pan et al. 2007; Zang et al. 2015). This uplift led to the rearrangement of drainage systems and migration of major river channels, isolating the species on opposite sides of the Altai Mountains. Specifically, Mongolian grayling (*T. brevirostris*) became isolated in the northern Mongolian Kobdo Basin, while Markakol grayling (*T. brevicephalus*) remained on the southern side of the Altai range. During this period, the Altai Mountains moved, with one end of the fault block forming a near‐upright tilted structure, and on the southern slope there is a continuous mountain massif. The asymmetric north–south topography created distinct environments for fish. The steep and short northern slope of the Altai Mountains lies adjacent to the large Mongolian Kobdo Basin. Mongolian grayling underwent divergent evolution within the species' poor lakes of the basin, and developed a phenotype that included well‐developed jaws, teeth, palatine and tongue as probable adaptations to harsher environmental conditions (Knizhin et al. 2008). Many mountainous regions globally exhibit noticeable disparities in drainage systems and ecosystems on either side, which are often attributed to the results of tilting movements. For instance, the formation of watersheds caused by mountain uplift further led to the rearrangement of river systems, which shaped the distribution patterns of grayling species in the Baikal Lake basin (Knizhin, Weiss, and Sušnik 2006; Koskinen, Nilsson et al. 2002). The rapid uplift of the Qinling Mountains has led to river capture‐related events, resulting in fish dispersion and the formation of isolated habitats in the north and south (Liu et al. 2014; Chen, Jiao, and Ni 2022).

One of the striking findings in this study is the differentiation between eastern and western groups of *T. brevicephalus*, We report two distinct geographic groups (A and B) in this area, Genetic divergence is observed between eastern (group B: Karaertis and Kayiertesi rivers) and western sides (group A: Kara‐Kaba, Akkaba, Kanas River, Burqin, Hemu and Crane rivers, and Chonghuer and Hongqi reservoirs), with the Crane River (KE) serving as a potential boundary between these genetically distinct lineages. Both mtDNA and microsatellite data corroborate this geographical pattern. The dating analyses yielded divergence times at 0.48 Ma (0.30–0.71 Ma), suggesting a Pleistocene split between the two geographic groups. We hypothesize that differences in these geographic groups are associated with the occurrence of glacial refugia, a concept supported by various theories. During the Pleistocene epoch, two spatially separated lineages of *Thymallus* in Lena River may have arisen through prevalence of a Polar continental shelf ice sheet during the Siberian Pleistocene, which isolated *Thymallus* populations in glacial refugia in the Lena Delta and middle reaches of Lena River (Weiss et al. 2006). In Europe, the European grayling (*Thymallus thymallus*) exhibits significant genetic differences over relatively short geographical distances, a phenomenon attributed to glaciation‐mediated processes (Gum, Gross, and Kuehn 2005; Koskinen, Sundell et al. 2002). Similarly, in North America, *T. arcticus* underwent genetic differentiation during a Pleistocene glacial period because of refugia formation (Redenbach and Taylor 1999; Stamford and Taylor 2004). During the continuous uplift of the Altai Mountains, the region experienced its first Quaternary glaciation. As the Altai Mountains froze, their southern slope was influenced by the MIS12 Burqin Glaciation (0.47 ± 0.051 Ma) (Devyatkin 1981; Jiang 2012). During a period of significant climate cooling, many glaciers formed, creating glacial refuges in river valleys for various organisms. This period coincides with the divergence time of the eastern and western lineages of *T. brevicephalus*. Evidence suggests that river valleys at the southern foot of the Altai Mountains served as such refugia during the Pleistocene ice ages, harboring species like the Green toad (*Bufo viridis* subgroup), *Clematis sibirica*, and *Larix sibirica* (Zhang et al. 2008; Zhang and Zhang 2012; Zhang, Zhang, and David 2014). It is also possible that *T. brevicephalus* sought refuge in these glacial refugia within the river valleys during that time.

However, while ecological barriers are often considered a primary driver of divergence, it is essential to recognize that other factors, such as behavioral isolation, or selective pressures, may have contributed to the observed genetic patterns. Comparative studies of freshwater fish in other regions provide insights into the possible mechanisms of genetic divergence. For instance, the studies on sea trout (*Salmo trutta*) in Estonian and Russian (Koljonen, Gross, and Koskiniemi 2014) and research on *S. trutta* (L.) in European rivers (Bekkevold et al. 2020) suggests that life history traits, such as short‐distance migration, natal homing, and reproductive isolation, can result in genetic divergence even in the absence of obvious physical barriers. Notably, the easternmost (Kayiertesi River) and westernmost (Kara‐Kaba River) sampling locations are separated by only 280 km. The genetic distances indicate a Moderate level of differentiation (FST > 0.05) between these populations. The genetic differentiation observed between eastern and western populations of *T. brevicephalus* at a relatively small geographic scale suggests a conservative behavioral pattern, consistent with the genus's typical life history traits. Specifically, grayling exhibit limited dispersal and strong reproductive migration behaviors, which can hinder gene flow between different geographic populations, and even within the same waterbody, leading to genetic differentiation (Gönczi 1989; Northcote 1995; Nykänen, Huusko, and Mäki‐Petäys 2001). Furthermore, phylogenetic analysis indicates that the upper Enisei grayling (*T. nikolskyi*), which coexists with *T. brevicephalus* in the Irtysh River system, exhibits a more distant genetic relationship compared to the closer genetic relationship between *T. brevicephalus* and Mongolian grayling (*T. brevirostris*). This highlights that the pattern of genetic differentiation is further accentuated at the species level.

### 4.2 Genetic Diversity and Expansion in Small Populations

Genetic diversity, shaped by natural processes throughout species evolutionary history, has been recognized by the IUCN as a crucial component of biodiversity. In this study, microsatellite markers consistent with prior research on three *T. brevicephalus* populations in Kazakhstan were used (Weiss, Grimm et al. 2020) to enhance comparability with existing studies. The Na, AR, HO, and HE values observed in this study are similar to the results of previous research in Kazakhstan. The genetic diversity observed in this study confirms existing information in the field. Takezaki and Nei (1996) suggested that the heterozygosity of naturally expanding populations should range between 0.5 and 0.8. The HO (0.583–0.677) and HE (0.772–0.876) values for *T. brevicephalus* in this study indicate that they are stable populations. However, the FIS values for the population suggest a certain degree of inbreeding in *T. brevicephalus*, hinting at a possible historical population bottleneck event, the mtDNA results indicate that *T. brevicephalus* falls into a group with a high level of haplotype diversity (Hd > 0.5) and a low level of nucleotide diversity (π < 0.005) (Grant, 1998), consistent with the genetic diversity pattern observed in *T. grubii*, *T. nikolskyi*, *T. brevirostris*, and *T. Thymallus* (Ma et al. 2012; Weiss, Grimm et al. 2020; Pettersen et al. 2021). This pattern suggests that during population expansion, the increase in population size leads to higher haplotype diversity, but nucleotide variation cannot accumulate in a short period, resulting in a founder effect. Consequently, the population may be subject to potential genetic drift. The Bayesian Skyline Plots (BSP) indicate that *T. brevicephalus*, particularly the western group, may have experienced extensive postglacial colonization from separate refugia. Mismatch distribution and neutrality tests also support evidence of population expansion.

These phenomena are likely associated with the interglacial periods that followed glacial epochs within the Central Asian region (Agatova et al. 2019; Bohorquez, Jimenez‐Ruiz, and Carling 2019; Herget et al. 2020). During the late Quaternary interglacial climate changes, rising temperatures, increased water flow, and glacial melt in the Central Asian region caused the breaching or overflow of moraine‐dammed lakes in mountain basins, resulting in multiple giant floods, including the Altai Great Flood event (Komatsu et al. 2015). The recurrent flooding in the Altai mountain valleys created favorable conditions for the population expansion of aquatic organisms in this region (Agatova et al. 2019; Weiss, Grimm et al. 2020).

As the boundary between the eastern and western regions, the presence of Crane River's haplotypes in grayling from both evolutionary geographic groups most likely reflects a scenario of secondary contact following postglacial expansion (Larson et al. 2013; Wen and Fu 2021). This contact zone (KE) contains haplotypes from both group A and B and exhibits higher levels of genetic diversity compared to other populations.

### 4.3 Conservation and Management Implications

Due to increasing human activities, *T. brevicephalus* populations have suffered a serious decline of over 50% due to overfishing in Ob‐Irtysh River headwaters (Weiss et al. 2021). Genetic diversity studies indicate that the species is at risk of reduced genetic diversity over time and may lack the ability to adapt to environmental changes. These findings highlight the urgent need for strengthened conservation and management efforts for *T. brevicephalus*. To safeguard the genetic resources and habitats of the grayling in the upper Irtysh River, which is listed as endangered in Kazakhstan and mistakenly identified as the Arctic grayling (*T. arcticus*) in China, the species has been included in China's national register of key protected wildlife, prohibiting its commercial capture.

Based on this study, we recommend reclassifying the currently listed Arctic grayling (*T. arcticus*) in Xinjiang, China, to reflect its true identity and implementing international cooperation in conservation efforts alongside Kazakhstan, given the shared population across the border in the vicinity of Lake Markakol. While recognizing the species as a whole, the observed genetic differentiation between eastern and western populations necessitates careful management, particularly concerning restocking efforts. To prevent genetic admixture, restocking should utilize fish specific to their respective eastern or western geographic group, with the Crane River acting as a natural boundary. Furthermore, the Crane River population, exhibiting characteristics of both geographic groups, could be considered a unique intermediate group and warrant separate conservation efforts. This approach safeguards the genetic integrity and unique evolutionary history of each lineage, ultimately preserving the overall health and diversity of the *T. brevicephalus*.

## Author Contributions

**Wenjie Peng:** formal analysis (lead), validation (lead), visualization (lead), writing – original draft (lead), writing – review and editing (lead). **Haoxiang Han:** conceptualization (supporting), investigation (equal), methodology (supporting). **Bo Ma:** conceptualization (lead), data curation (lead), formal analysis (equal), funding acquisition (lead), investigation (lead), resources (lead), writing – original draft (equal), writing – review and editing (equal).

## Ethics Statement

This study has passed the application for ethical review of experimental animal welfare at the Heilongjiang Fisheries Research Institute (20190820–001).

## Conflicts of Interest

The authors declare no conflicts of interest.

## Supporting information


**Figure S1.** Net pairwise distances (uncorrected p‐distances) based on CR (below diagonal) and Cyt b + CR (above diagonal), geographic group distribution, as shown in figure 1, is color‐coded.

**Figure S2.** Bl and ML phylogenetic reconstructions using the CR sequences. Branch support values indicate Bayesian posterior probabilities (the left side of the node values) and maximum likelihood values (the right side of the node values).

**Figure S3.** Bl and ML phylogenetic reconstructions using the Cyt b + CR sequences. Branch support values indicate Bayesian posterior probabilities (the left side of the node values) and maximum likelihood values (the right side of the node values).

**Table S1.** Microsatellite loci.

## Data Availability

The datasets generated during and analyzed during the current study are available in the GenBank (PP425404—PP425725).
