# Supplementary figures and images for: Genetic Structure and Phylogeographic Divergence of Thymallus brevicephalus in the Ob‐Irtysh River Headwaters
Source: Ecol Evol. 2024 Oct 11;14(10):e70422. doi: 10.1002/ece3.70422 (PMC11470089; doi:10.1002/ece3.70422)

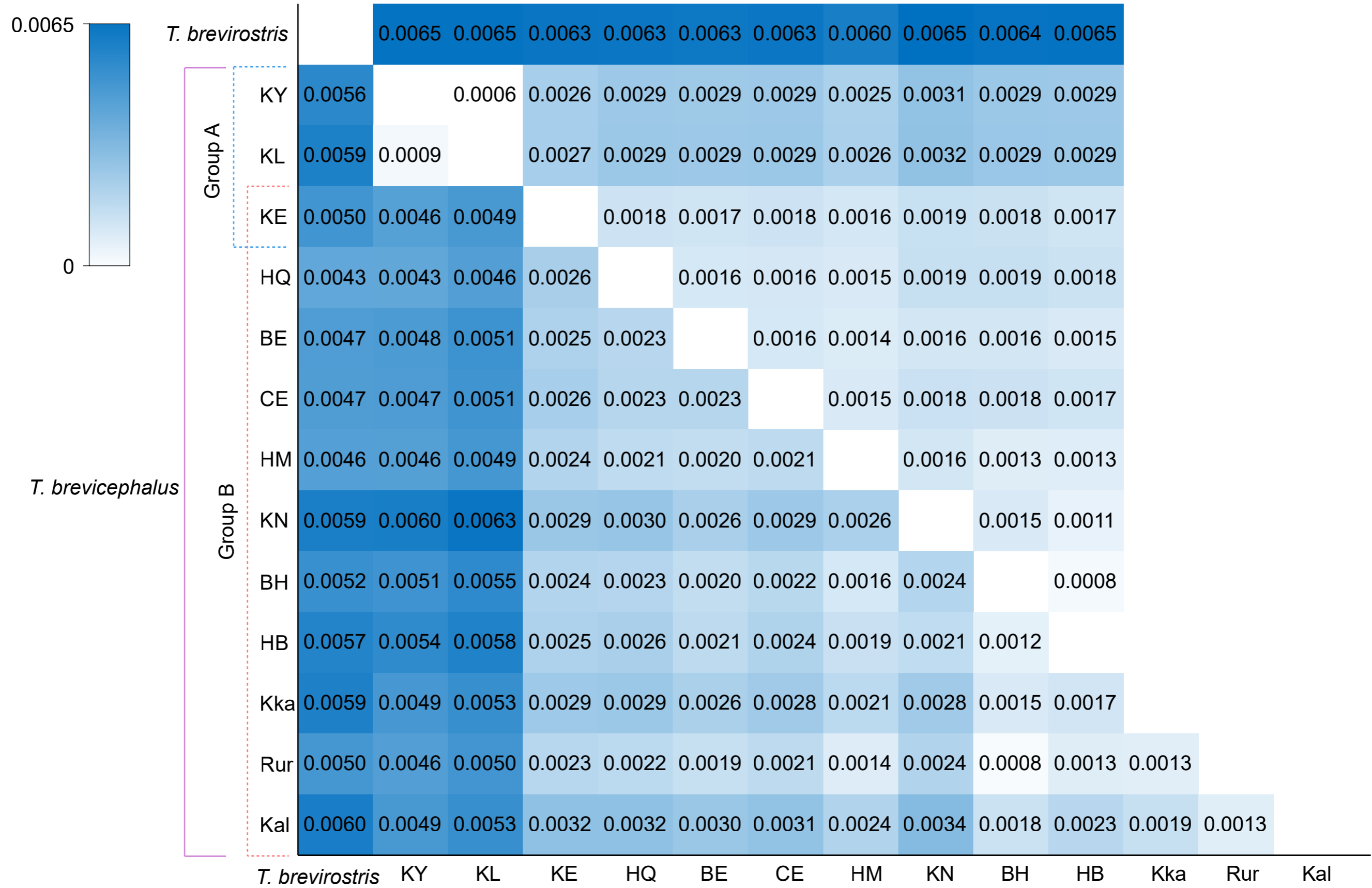

Supplement: Supplementary file 1 — Figure S1. Net pairwise distances (uncorrected p‐distances) based on CR (below diagonal) and Cyt b + CR (above diagonal), geographic group distribution, as shown in figure 1, is color‐coded. [file ECE3-14-e70422-s002.pdf]

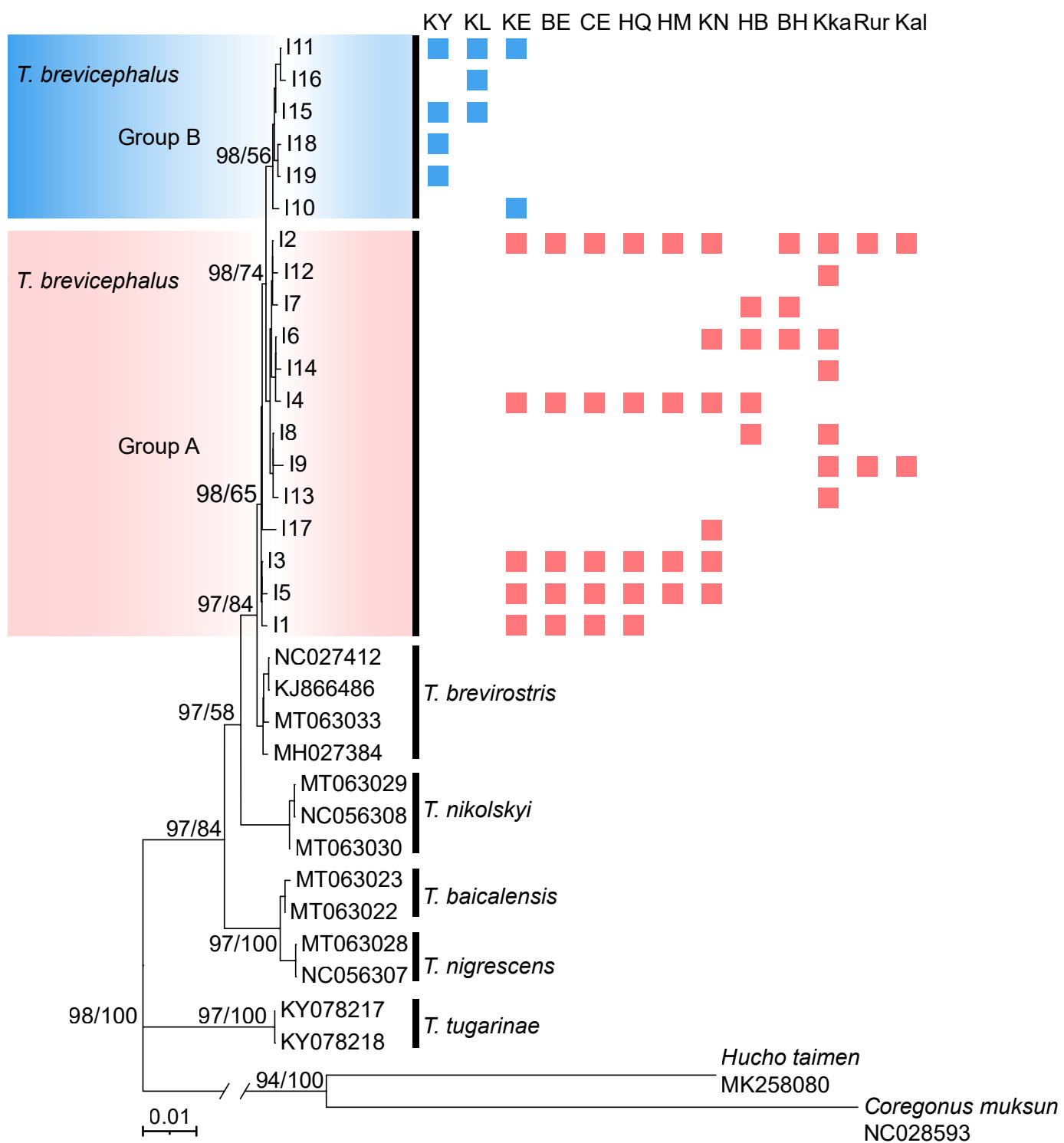

Supplement: Supplementary file 2 — Figure S2. Bl and ML phylogenetic reconstructions using the CR sequences. Branch support values indicate Bayesian posterior probabilities (the left side of the node values) and maximum likelihood values (the right side of the node values). [file ECE3-14-e70422-s001.pdf]

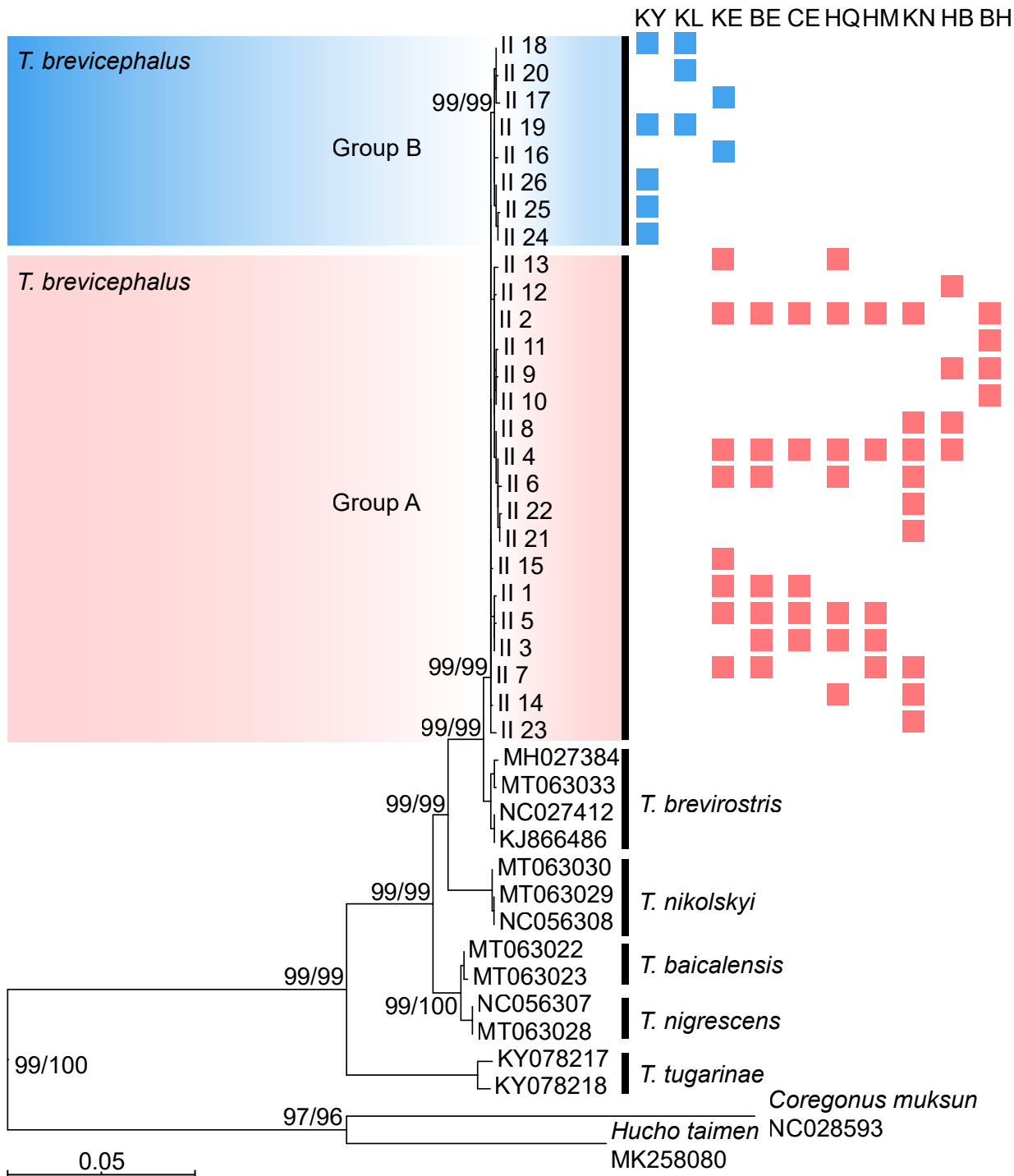

Supplement: Supplementary file 3 — Figure S3. Bl and ML phylogenetic reconstructions using the Cyt b + CR sequences. Branch support values indicate Bayesian posterior probabilities (the left side of the node values) and maximum likelihood values (the right side of the node values). [file ECE3-14-e70422-s003.pdf]
