# Supplementary material for: Genetic Structure and Phylogeographic Divergence of Thymallus brevicephalus in the Ob‐Irtysh River Headwaters
Source: Ecol Evol. 2024 Oct 11;14(10):e70422. doi: 10.1002/ece3.70422 (PMC11470089; doi:10.1002/ece3.70422)
Supplement: Supplementary file 4 — Table S1. Microsatellite loci. [file ECE3-14-e70422-s004.docx]

| **Table S1** Microsatellite loci | | | |  |
| --- | --- | --- | --- | --- |
|  | Locus | Repeat motif | Primer sequence (5′–3′) | Ta (°C) |
| Single | ClaTet1 | (GACA)13 | F: GAGCCCATCATCACTGAGAAAGA | 60°C |
|  |  |  | R: CTGCTACCCACAAACCCCTG |  |
| 4Plex | BFRO004 | (GT)11 | F: GCTCCAGTGAGGGTGACCAG | 58°C |
|  |  |  | R: AGGCCACTGATTGAGCAGAG |  |
|  | BFRO010 | (AC)17 | F: GGA CGG AGC CAG CAT CAC | 58°C |
|  |  |  | R: GTTTCTTGATTTCATAATCAGGTCAATAGTCAT |  |
|  | Tar103 | (ATCC)7TCC(ATCC)14 | F: CAGTCGGGCGTCATCACGGGGATCAATAAAGTATCC | 58°C |
|  |  |  | R: CTTCACTGTCGCTGTGAGTAC |  |
|  | Tth445 | (GATA)20 | F: TGA CGG CTA CAG GAA TTGT | 58°C |
|  |  |  | R: GTTTCTTCCACAGAGGGTTCTACATTG |  |
| 5Plex | Tar100 | (CTTT)5CTTC(CTTT)18 | F: CAGTCGGGCGTCATCATTTGGATGTGTCAGACCTG | 58°C |
|  |  |  | R: GAGAAAGCAAGGAGAAATCAC |  |
|  | Tar101 | (CTTT)22 | F: CAGAGCACACCAAGCAGAG | 58°C |
|  |  |  | R: GTTTCTTAGGGCAAGTCATTCCAGTC |  |
|  | Tar110 | (TAGA)30 | F: GCAATAACAATTCCATGAGAAG | 58°C |
|  |  |  | R: GTTTCTTCTCCTCTGATTCCAAGAAATG |  |
|  | Tar112 | (TATC)7 | F: CAGTCGGGCGTCATCACCTGGGAATCAACAAAGTATC | 58°C |
|  |  |  | R: AGGAGGTTCAGTGAGTGTTTC |  |
|  | Tth313 | (GAGT)22 | F: AAACCAGTCCAAGCGAGAG | 58°C |
|  |  |  | R: GTTTCTTCTCCTGTTTATCACATGA |  |
